# Correlations of the “Work–Family Conflict” With Occupational Stress—A Cross-Sectional Study Among University Employees

**DOI:** 10.3389/fpsyt.2020.00134

**Published:** 2020-03-18

**Authors:** Lucia Jerg-Bretzke, Kerstin Limbrecht-Ecklundt, Steffen Walter, Jennifer Spohrs, Petra Beschoner

**Affiliations:** ^1^Department of Psychosomatic Medicine and Psychotherapy, Medical Psychology, University Medical Centre, Ulm, Germany; ^2^Department of Psychosomatic Medicine and Psychotherapy, Ulm University Medical Centre, Ulm, Germany

**Keywords:** work–family conflict, working conditions, occupational stress, effort–reward imbalance, mental health, university staff

## Abstract

**Background:** The working conditions at universities and hospitals are reported to be stressful. Several national and international studies have investigated occupational stress in hospitals. However, scientific studies at colleges and universities addressing psycho-social stress factors and their potential consequences are scarce. In this context, the consequences and correlations of the factor of work–family conflict, in particular, are currently uninvestigated. The aim of our study was to assess data on psychosocial stress in the context of the compatibility of work and family.

**Methods:** Data were gathered through a cross-sectional-study, *N* = 844 (55% female, 41% male), on university staff (42.3% scientists, 14.3% physicians, 19.4% employees in administration, and 19.3% employees in service). Participants filled out questionnaires to provide their personal data and details of their work and private life conditions. For this purpose, we used the Work–Family and Family–Work Conflict Scales, Effort-Reward Inventory and Overcommitment Scale (ERI, OC), Patient Health Questionnaire (PHQ-4), short-form Maslach Burnout Inventory (MBI), and questions on their subjective health. Statistical analyses were performed using SPSS 22.

**Results:** We found high levels of stress parameters in the total sample: extra work (83%), fixed-term work contracts (53%), overcommitment (OC, 26%), Effort-Reward Imbalance (18%, ERI Ratio > cut-off 0.715), work–family conflict (WFC, 35%), and family–work conflict (FWC, 39%). As hypothesized, we found significant correlations of both WFC and FWC with psychosocial work strain (ERI Ratio) as well as overcommitment (OC). Mental and somatic health parameters also had a significant positive correlation with WFC and FWC. Using a regression analysis (*N* = 844), we identified WFC as a predictor of burnout, while emotional exhaustion, extra work, and overcommitment could be identified as predictors of WFC and FWC.

**Discussion:** The results of our study point toward deficits in the compatibility of work life and private life in the work fields of science, colleges, and universities. Furthermore, we found indicators that work–family conflicts (interrole conflicts) have an impact on mental and somatic health. These work–family conflicts should be targets for preventions and interventions with the aim of improving the work-life balance and mental and somatic wellbeing of employees.

## Introduction

In our globalized and increasingly digitalized occupational environment, employees are exposed to more and more arduous occupational conditions and occupational stress. This has an impact on their health and well-being (1). Particularly affected are colleges, universities, and university hospitals, since they are confronted with a global competitive environment shaped by the pressure of competition for research funds, personal resources, and students. Combined, this leads to high demands regarding flexibility, mobility, and spatiotemporal adaptability (2). Furthermore, the increasing number of students, which is not accompanied by an adequate increase in teaching professionals (3), educational reforms, harsher competitive conditions, and economization pose new framework requirements in academia (4). In recent years, these structural changes have led to exponential work intensification and occupational stress for university employees (5, 6). For German scientific researchers, the working conditions can be considered precarious. In 2015, nine out of ten university employees were employed with fixed-term contracts, according to a newspaper report (7). Scientific associates are highly qualified and motivated but underpaid, with a considerable amount of unpaid overtime, non-transparent as well as uncertain career perspectives, all in combination with a very high variety of tasks and occupational stress (8, 9). Yet another affected occupational area is university medicine with its associated university hospitals. Multiple studies describe the working conditions in hospitals as arduous and wearisome, demonstrate the link to health impairments, and indicate the working conditions as a central factor determining work performance (10, 11). The weekly hours of work, complemented by shift work, stand-by duty, special services, and time pressure, are considered to be the main stress factors (12, 13). Furthermore, the mental workload in the occupational area of university medicine is extremely high, and the already stressful working conditions are aggravated by aspects such as multi-tasking, high informational complexity, and constant deadline constraints, as well as the pressure of gaining qualifications and a limited scope of action (14).

For a long time, psychiatric disorders have shown the greatest increase in the causation of temporal and long-term absence from work (15). According to a study of the Techniker health insurance fund (1), “work” turns out to be the number one stress factor in Germany and is consequently associated with various consequences of strain, for the individual as well as for the economy. For that matter, subjectively experienced stress, particularly psychosocial occupational stress, poses a high-risk factor for mental and physical health. Mentioned in this regard are an increased susceptibility to infections, cardiovascular diseases, diseases of the musculoskeletal system, depression, anxiety disorders, burnout, and a subjectively experienced poor state of health (1, 16). Related studies indicate differences between the various occupational groups. For instance, national, and international studies have shown that deficient or impairing conditions and psychosocial strain, particularly in the context of medical work, share joint responsibility for mental and physical health discomfort (11, 17). The demands on medical practitioners are explicitly displayed in higher numbers of anxiety and depressive symptoms (18). Dech (5) refers to the high mental workload, which can be particularly acute for scientists, leading to adverse psychological health effects. In this context, he describes multi-tasking, high informational complexity, and constant deadline constraints as well as pressure to gain qualifications as the causes for multiple-stress syndromes, reactions to excessive demands, burnout, and substance abuse.

Different models and instruments exist for assessing psychosocial occupational stress with regard to the impact of its stress-related consequences. For our study, the perspective is to integrate this psychosocial occupational stress into a model of increased risk for physical, mental, and health disorders, which is best covered by the framework of the professional gratification crisis by Johannes Siegrist. His model assesses the experience and perceptions of employees with regard to their effort and the adjunctive reward with the ERI (Effort-Reward Imbalance) questionnaire, and the tendency to overcommit with the Overcommitment questionnaire (19). The ERI ratio is deemed to be an independent predictor for the increased emergence of disorders, for example psychological disorders such as depression, substance abuse, coronary diseases, psychosomatic disorders, and burnout (19–22). Siegrist and Siegrist (19) found that high values for the ERI ratio were especially prevalent in personal, non-productive industries, for example, medical staff. They also discovered that men are more frequently affected than women.

The compatibility of family and work is a challenge that concerns occupational groups. A work–family, or interrole, conflict is defined as an impairment of the different roles (occupational and family-related) in the exertion of their duties (23). The effect can be bidirectional; depending on the direction of the conflict, it is considered a “work-(to)-family conflict” when the occupation impairs the familial or private life, and, vice versa, a “family-(to)-work conflict” when the private life influences the occupation (24, 25). Various studies have furnished evidence for the fact that occupational stress has higher implications on the family than contrariwise (26). Occupational groups with a high load of labor time, irregular working hours, and unstable working conditions, especially, suffer from this work–family conflict. Physicians and the scientific staff of universities and university hospitals fall into this type of occupational group (9, 11, 27). Studies in varied occupational areas mention primarily high occupational stress, long working hours, excessive overtime, low temporal flexibility, deficient social support and recognition as reasons for the difficulties with balancing work and family life (28, 29). Correlations between an interrole or work–family conflict and mental and physical health have been demonstrated by various studies (30, 31). In 1990, Firth-Cozens (32) reported a significant correlation between a sensed conflict and the presence of depression, which is relevant to the compatibility of occupation and family.

Previous studies on the topic of occupational stress and health stem mainly from the economic and health sectors, rarely from institutions such as universities and colleges. Notably, a comparison between the individual occupational areas at universities is missing. Furthermore, to date, research on occupational stress has neglected the influence of the factor compatibility of occupation and family on the individual occupational stress and health of the employee (26).

The work–family or interrole conflict is considered a predictor of health determinants, according to literature/previous research. The same can also be assumed the other way around. Based on this background, we have deliberately chosen an exploratory approach to investigate the influence of stress and health factors on interrole conflict.

The aim of this study was to assess data on the work–family or interrole conflict in the context of psychosocial occupational stress and health in a university context and to illustrate their indications of interdependencies.

## Methods

The presented results are part of an online survey, which was approved by the ethics committee of Ulm University, application number 246/11, on the 14th of November 2011. Participation was voluntary, and all participants signed an informed consent form. For the quantitative data acquisition, participants filled out demographic questionnaires and replied to individual questions regarding their work situation, such as their contract of employment, overtime, and related occupational stress, etc.

To gather information on the interrole or work–family conflict, meaning the impact that work has on family life and vice versa, we used the Work–Family and Family–Work Conflict Scales (WFC, FWC) (24). The questionnaire has two subscales consisting of five items each. The first one investigates the conflicts that work life may impose on families (WFC), and the second subscale measures the inverse effect (FWC). For example, an item of the WFC scale is: “The requirements at work collide with my private and familial life,” and an item of the FWC scale is: “The requirements on the part of my family collide with my professional tasks.” Items were scored on a five-point Likert scale ranging from 1 = “no, not at all” to 5 = “yes, exactly. The higher the added scores, the higher the conflict potential. Cronbach's Alpha is 0.88 for the WFC scale and 0.86 for the FWC scale (24).

The Effort-Reward Imbalance Questionnaire (ERI) (33) was deployed to capture the psychosocial workload. The instrument indicates possible imbalances between effort and reward in occupations. For this purpose, six questions coded on a five-point Likert scale, concerning the stressful aspects and 11 items concerning the rewarding aspects of work were assessed. The resulting ERI ratio indicates the proportions of effort and reward. The higher the effort–reward quotient, the higher the disproportion between effort and reward. Lehr and colleagues (34) classify an effort–reward imbalance as ERI ratios equal to or higher than 0.715.

The overcommitment scale (OC) (33) measures excessive performance motivation and tendency to overcommit by means of six items on a four-point Likert scale.

To assess health-related parameters, first, the subjectively experienced physical fitness was determined by means of one question (six-point Likert scale, 1 = very good to 6 = deficient) (35). The Patient Health Questionnaire (PHQ-4) is a short questionnaire with a four-point Likert scale that addresses symptoms of depression and/or anxiety by means of two items each (36).

Burn-out syndrome describes a state of exhaustion that is accompanied by emotional exhaustion (EE), reduced feelings of personal accomplishment (PA), and depersonalization (cynicism) (DP) (37). One of the internationally most elaborated instruments for measuring this is the Maslach Burnout Inventory (MBI) (38). The authorized German translation of the MBI by Büssing and Perrar was shortened by Prof. Dr. Jürgen Glaser (University of Innsbruck) (Glaser, manuscript in preparation). This short version assesses only two factors of the MBI: emotional exhaustion (EE) and depersonalization (cynicism) (DP) with three questions each, coded on a six-point Likert scale.

The statistical analysis of the data generated with the online survey tool EVASYS was carried out with the statistical program SPSS 22. Comparisons between subgroups, for example, males versus females, were analyzed using Chi-Square tests. Further, we conducted correlation analyses to calculate the interdependencies between the different instruments: Pearson's method for nominally and ordinally scaled items, and Spearman's method for interval-scaled items. To determine the linear correlations, we conducted multiple regression analyses using stepwise regression analysis (39).

The study as a whole had a theoretical or literature-guided basis. Due to a lack of longitudinal data, the regression analyses were conducted with an exploratory approach. The factors used were chosen on the basis of pre-calculated significant correlations.

These baseline calculations have already been used in the context of a habilitation thesis.

## Results

### Demographics

The total sample of *N* = 844 university employees consisted of 55.5% (*N* = 468) women and 40.9% (*N* = 345) men; 3.7% (*N* = 31) did not specify a gender. Split into occupational groups, 42.3% (*N* = 357) were scientific researchers, 14.3% (*N* = 121) physicians, 19.4% (*N* = 164) administrative employees, and 19.3% (*N* = 162) employees of various services, such as of technical, functional, or central services and/or laboratory technicians, janitors, etc. The age distribution of the total sample had its focal point in the age group between 30 and 40 years old. As the highest educational attainment, 79.4% (*N* = 670) of the total sample stated to have a high school degree, and 73.1% (*N* = 617) had a university degree. In terms of family demographics, 76.1% (*N* = 642) were in a partnership, and 49.9% (*N* = 421) had children (Table 1).

**Table 1:** Sociodemographic data.

	**Total sample**	
***N***	844	
Women	468	(55.5%)
Men	345	(40.9%)
**Age**		
<30 years	188	(22.3%)
30–40 years	284	(33.6%)
41–50 years	214	(25.4%)
> 50 years	153	(18.1%)
**Highest educational attainment**		
Lower secondary school	31	(3.7%)
Secondary school	120	(14.2%)
High school	670	(79.4%)
Other	19	(2.3%)
**University degree**		
Yes	617	(73.1%)
No	214	(25.4%)
**Family status**		
In a partnership	642	(76.1%)
Single	189	(22.4%)
**Children**		
Yes	421	(49.9%)
No	417	(49.4%)

For administrative reasons, the data for the staff working in the medical department had to be collected separately. Thus, there were two response rates: the occupational group of research scientists, administrative employees, and employees of various services (33.6%; *N* = 537), and employees of the medical department (22%; *N* = 307).

### Working Conditions

Out of the 844 participants interviewed, 68% (*N* = 574) declared that they worked full-time, and more than half of the sample (*N* = 449; 52.9%) had fixed-term contracts. Overtime accounted for 83.3% (*N* = 698) of the respondents, and 19.7% (*N* = 166) of the sample were “always and frequently” stressed (Table 2).

**Table 2:** Overtime and stress due to overtime.

	**Total sample *N* = 838**	
Overtime, yes:	698	(83.3%)
Stress due to overtime:	*N* = 732	
Always	38	(4.5%)
Frequently	128	(15.2%)
Sometimes	325	(44.4%)
Rarely	182	(24.9%)
Never	59	(8.1%)

### Work–Family Conflict

The impact of the professional work on the familial life and the impact of the familial life on the professional work was assessed with the Work–Family Conflict Scale and the Family–Work Conflict Scale (24). The means of the WFC scale (*M* = 14.34; *N* = 813; *SD* = 5.81) and of the FWC-scale (*M* = 10.08; *N* = 800; *SD* = 4.40) were only slightly (ns) below the standard values as defined by Netemeyer and colleagues (WFC Scale: 16.69; FWC Scale: 10.68). These standard values can be used as cut-off values, and more than one third (35.3%) of the respondents (*N* = 813) scored higher than the cut-off for WFC and close to 40% (*N* = 800) scored higher than the cut-off for the FWC, indicating a work–family or, respectively, an interrole conflict. In both categories, men suffered more often (but not significantly) from this conflict than women.

### Psychosocial Occupational Stress and Tendency to Overcommit

The psychosocial occupational stress was assessed with the model and measuring instrument Effort-Reward Imbalance (ERI) developed by Siegrist. With a mean of *M* = 10.32 (*N* = 799; *SD* = 3.32), the effort value of the sample was still lower (n.s.) than the mean of the norm sample (*M* = 11.57). However, the average reward value (*M* = 37.04; *N* = 668; *SD* = 6.45) was also lower (*p* = 0.000) than the norm sample (*M* = 46.71). Thus, the ERI ratio, as a value for the proportion between work effort and work-related reward, was used as an index for the psychosocial occupational stress. Here, high effort and little reward go along with a high ratio. A value higher than the cut-off value of 0.715 (34) is considered as posing a risk to health.

On average, the total sample did not reach this cut-off (*M* = 0.56; *N* = 649; SD = 33), but almost a fifth of the sample (17.1% of *N* = 649) scored higher than the cut-off, with considerably more men (18.7%, total *N* = 268) than women (15.7%, total *N* = 356) suffering from this imbalance. For this part of the sample, an imbalance between effort and reward was apparent, meaning that the affected employees subjectively perceived a higher effort in relation to a smaller reward. According to the Effort-Reward Imbalance Model, this imbalance may lead to stress responses and eventually to health impairments.

The Overcommitment Scale measures the individual tendency to overcommit professionally. In connection with an excessive tendency to overcommit, an increased amount of stress reaction and an increased risk of illness is assumed. The mean of the overcommitment scale, *M* = 14.4 (*SD* = 3.20; *N* = 806), was also within the normal range. The cut-off value of 16 was exceeded by more than a quarter (26%) of the respondents (*N* = 807), and women were considerably more often affected than men.

### Health and Burnout

The subjectively experienced state of health of the sample was between good (2) and satisfactory (3) (*M* = 2.56; *SD* = 1.02; *N* = 828). Of the participants, 43.1% (*N* = 830) described their health status as satisfactory (3), and 42.2% of men (Total *N* = 339) and 44.3% of women (Total *N* = 461) described it as worse (4 = sufficient; 5 = poor; 6 = deficient).

Data on mental health were assessed with the Patient Health Questionnaire (PHQ-4), which contains two questions each to screen for a depressive disorder according to the DSM-IV and a generalized anxiety disorder (36). Higher values indicate higher troubles associated with the depressive or anxiety disorder. A mean higher than 2.5 (cut-off) is considered to be critical in terms of the measured symptoms. With *M* = 1.45 (*SD* = 2.21; *N* = 822), the total sample did not reach this cut-off. However, 7% of the sample (*N* = 823), 6.4% of women, and 7.1% of men scored higher than the cut-off.

The short version of the MBI (Glaser, manuscript in preparation) was used to assess the burnout-related symptoms. The scaling of the MBI is 0 = never, 1 = very rarely, 2 = rather rarely, 3 = sometimes, 4 = rather often, and 5 = very often. The mean of the sample at hand was M = 2.5 (*SD* = 0.99; *N* = 832), which falls between the scale values “very rarely” and “rather rarely.” The average values for women (*M* = 2.48; *SD* = 0.96; *N* = 462) and men (*M* = 2.53: *SD* = 1.02; *N* = 340) show no significant difference.

### Correlations With Work–Family Conflict

We calculated correlations between the two parameters of the work–family conflict and the occupational stress and the health parameter, respectively. Firstly, we found high positive correlations between the conflictual influence of work on familial life (WFC) and both occupational stress as measured by the ERI ratio and the tendency to overcommit. Furthermore, we found significant high correlations between WFC and the item “overtime is stressful”: the more often overtime was perceived as stressful, the higher the work–family conflict (the negative correlations stem from the direction of the scaling of the item “overtime is stressful”) (Table 3).

**Table 3:** Correlations of the Work–Family Conflict Scale and Family–Work Conflict Scale with occupational stress (ERI Ratio), overcommitment (OC), and whether overtime is stressful.

**Work–Family Conflict Scale with…**	**Occupational stress (ERI Ratio)**	**Overcommitment (OC)**	**Overtime is stressful^**+**^**
Total sample	*r* = 0.600^**^ *N* = 634	*r* = 0.508^**^ *N* = 786	*r* = −0.562^**^ *N* = 709
Family–Work Conflict Scale with…			
Total sample	*r* = 0.422^**^ *N* = 629	*r* = 0.334^**^ *N* = 775	*r* = −0.337^**^ *N* =697

*+Scaling 1 = “always”, 2 = “frequently”, 3 = “rarely”, 4 = “never”*.

** *Correlations are significant at a level of 0.01 (two-sided)*.

* *Correlations are significant at a level of 0.05 (two-sided)*.

A weaker connection was found for the correlation (*p* = 0.000) of the FWC-scale with the ERI ratio, overcommitment, and the item: “overtime is stressful.” The more intense the occupational stress was perceived to be, the higher the influence of the family on the professional life was experienced to be (Table 3).

The correlations between the health parameters “physical and mental health,” burnout, and the WFC and FWC scales were also significant. Here, the correlations between WFC and PHQ-4 and burnout were highest, and FWC correlated most strongly with burnout (Table 4). A weaker but still significant correlation (*p* = 0.000) could be found between the subjectively experienced physical health and the FWC and WFC scales (Table 4).

**Table 4:** Correlations of the Work–Family Conflict Scale/Family–Work Conflict Scale with physical health, mental health, and burnout.

**Work–Family Conflict Scale with…**	**Physical health^**+**^**	**Mental health (PHQ-4)**	**Burnout (MBI)**
Total sample	*r* =0.178^*^ *N* = 806	*r* =0.417^**^ *N* = 800	*r* =0.497^**^ *N* = 810
Family–work Conflict Scale with…			
Total sample	*r* =0.206^**^ *N* = 793	*r* =0.259^**^ *N* = 775	*r* = −0.329^**^ *N* =697

** *Correlations are significant at a level of 0.01 (two-sided)*.

* *Correlations are significant at a level of 0.05 (two-sided)*.

### Interdependencies of the Work–Family Conflict

Multiple regression analyses were calculated to analyze the functional interdependencies between the work–family or interrole conflict (WFC, FWC) as variables of interest and the occupational stress and health parameters. For the regression analysis of WFC, a corrected *R*^2^ = 0.313 was calculated. Thus, 31% of the variance of the work–family conflicts can be explained by the predictors burnout (factor: emotional exhaustion) (β = 0.278; *p* = 0.000), the sample (affiliation to the university or the university hospital) (β = −0.185; *p* = 0.000), “overtime is stressful” (β = −0.148; *p* = 002) (the negative prefix is due to the direction of the scaling of the item – “always” to “never”), gender (β = −0.132; *p* = 0.001), occupational area (β = −0.111; *p* = 0.014), and overcommitment (β = 0.102; *p* = 0. 031) (Table 5).

**Table 5:** Results of the regression analysis for the Work–Family Conflict Scale.

	**β-coefficient**	***t*-value**	***p***
(constant)		4.17	0.000
Burnout (MBI):Emot. Exhaustion pfung	0.278	5.68	0.000
Affiliation (1 = UU /2 = UK)	−0.185	−4.12	0.000
Overtime is stressful^+^	−0.148	−3.14	0.002
Gender (1 = m/2 = f)	−0.132	−3.44	0.001
Occupational area^*^	−0.111	−2.47	0.014
Overcommitment (OC)	0.102	2.16	0.031

*F_(6.498)_ = 39.262; R =0.567; corrected R^2^ = 0.313*.

*^+^Scaling: 1 = “always”, 2 = “frequently”, 3 = “rarely”, 4 = “never”*.

* *Occupational area 1 = “Physicians”, 2 = “Scientific researchers”, 3 = “Administrative employees", 4 = “Employees of various services”*.

The corrected *R*^2^ of the regression analysis with the variable of interest family–work conflict scale (FWC) was *R*^2^ = 0.154. The predictors explaining the variance for this scale were: “presence of children” in own household (β = −0.266; *p* = 0.000), followed by “overtime is stressful” (β = −0.199; *p* = 0.000), burnout (β = 0.168; *p* = 0.000), age (β = −0.134; *p* = 0.003), and gender (β = −0.084; *p* = 0.044) (Table 6).

**Table 6:** Results of the regression analysis for the Family–work conflict scale.

	**β-coefficient**	***t*-value**	***p***
(Constant)		12.58	0.000
Children^*^	−0.266	−5.87	0.000
Overtime is stressful^+^	−0.199	−4.18	0.000
Burnout (MBI)	0.168	3.53	0.000
Age	−0.134	−2.97	0.003
Gender (1 = m/ 2 = f)	−0.084	−2.02	0.044

*F_(5.494)_ = 19.208; R = 0.403; corrected R^2^ = 0.154*.

*^+^Scaling: 1 = “always”, 2 = “often”, 3 = “rarely,” 4 = “never”*.

* *Occupational area 1 = “Physicians”, 2 = “Scientific researchers”, 3 = “Administrative employees”, 4 = “Employees of various services”*.

## Discussion

The aim of our study was to collect cross-sectional data on the compatibility of professional life and family with regard to the psychosocial occupational stress and health in a university environment and to present their interdependencies. The response rates for a complete socio-scientific survey can be considered “good”. Even though it was not possible to test for representativeness due to missing comparative data, the data at hand can be used as an example to display the load situation at German universities based on the sample size and the response rate.

Even though the mean survey scores for the applied questionnaires concerning the occupational stress and health of the total sample were within the normal range, the study shows a substantial percentage of employees with above-average stress levels: 83% of the total sample (*N* = 698) worked overtime, and almost 20% felt “always and frequently” stressed by this. Almost 18% of the sample (*N* = 649) scored >1 in the ERI ratio, the indicator for occupational stress, and approximately a quarter (26%) of the sample (*N* = 807) showed an excessive overcommitment, posing a potential risk to their health.

An effort–reward imbalance and related professional gratification crises frequently arise for employees who find few alternative workplaces and for employees in competitive environments (19). University employees often encounter both situations. Due to their specializations, they rarely find alternative workplaces, for example, in free enterprises. Additionally, scientists and physicians are constantly confronted with a high workload and competitive pressure, and their working environments can be described as highly competitive. The imbalance between work effort and the received reward (ERI ratio), as defined by the ERI model, is sometimes evoked by a single disappointing event in the professional context (for example, a refused promotion, dismissal, degradation, etc.) However, more frequently, it is the result of long-standing re-occurring frustrations in the work context, which are accepted, no longer reflected upon, often trivialized, or even become part of the daily routine (40). The working conditions of the university employees alone, namely fixed-term contracts and overtime, offer reason for disappointment and frustration, but various daily stressors come in addition. In the medical working context, for example, the limited budgets that need to be adhered to and the administrative load can be stressful and frustrating. For scientific researchers, increasing competition, quantitative and qualitative pressure to publish, and high requirements for acquiring third-party funds are a daily stressor. Siegrist et al. (33) state that the ERI ratio is often intensified by intrinsic factors, especially when the professional coping mechanisms are dictated by excessive overcommitment. This maladministration is particularly grave for the affected employees, since the refused recognition or reward in this context can be considered a violation of the norm of social reciprocity. This suboptimal combination accounts for more than a quarter (26%) of the employees (*N* = 807). Especially in the scientific and medical occupational field, the consequences of this stress for performance and productivity are serious. For instance, for physicians who are exposed to a high load of psychosocial stress, the quality of the medical care decreases considerably (41, 42). To enable professional, high-level patient care, it is necessary to reduce the workload. High levels of stress in the form of a high ERI value are associated with different health impairments. Specifically, when exceeding the cut-off value of the ERI ratio, the risk for diabetes (43) and arteriosclerosis (44) increases. Furthermore, the risk for coronary heart disease doubles (45), and the prevalence of chronic diseases is increased (46). For overcommitment values above the cut-off, cardiovascular risks have been reported (47, 48). Additionally, multiple psychological implications have been found for an ERI, for example, sleeping disorders (49), alcohol addiction (50), substance abuse (51), and depression (52, 53).

The descriptive results of the health parameters fall widely within the normal range. Of the respondents, 56.9% (*N* = 860) described their state of health as very good or good. This result is below the statistics of the “labor time report” (54), where 62% of the interviewees described their state of health as very good and good. On the other hand, 43.1% of the participants (*N* = 860) described their state of health as merely satisfactory (3) and worse (4 = sufficient, 5 = poor, 6 = deficient). This should definitely be reason for interventions and preventive measures to increase the individual physical health of the employees.

In our study, the parameter “mental health” was operationalized by the survey instrument PHQ-4, which assesses symptoms of depression and anxiety (36). Anxiety disorders and depression present a life-time prevalence of 15–17% and are among the most frequent psychological diseases (55). In the present study, 7% of all the interviewed university employees (*N* = 823) presented a PHQ-4 score higher than the cut-off value of 2.5. Thus, they have “almost every day” “little of no joy or interest in their activities” and are “not capable of stopping brooding (ruminating or controlling their worries).” This presents a score that allows an initial suspicion for a depression or anxiety disorder. In the research literature, the reasons for this are mainly working conditions and related work–family conflict. Even though a tentative diagnosis can only be made for a comparably small percentage of the respondents, the stress impairments may be particularly grave for the university medical group. Impairments in this group may negatively impact the quality and quantity of the patient care (57). Additionally, since occupational stress is often linked to increased absenteeism, many absent days and high fluctuations may consequentially lead to high costs (56, 58).

There is evidence for gender differences in the prevalence of psychosocial occupational stress (1, 19). However, we could not identify significant gender differences for the measured parameters occupational stress (ERI ratio) and overcommitment (OC), nor for the parameters (stressful) working conditions, fixed-term contracts, overtime, or mobbing. However, it remains unclear why this is the case. For the parameter “subjective evaluation of the state of health,” we expected men to rate their physical state of health as higher than women, and we expected women to suffer more frequently from symptoms of depression and anxiety (1, 59). For the total sample of university employees, we could demonstrate that men indeed rated their physical health as better than women; however the difference was not significant. The PHQ-4 for depression and anxiety showed significant gender differences. Here, women display higher average scores. This is in line with the results of a study conducted by Techniker health insurance (1), which concludes that companies and institutes should focus primarily on women in their efforts to prevent mental suffering. Gender differences in the compatibility scales (WFC, FWC) will be discussed subsequently.

The compatibility of work and family is a central socio-political subject, especially due to the fact that the demographic changes and the resulting shortage of specialists can only be compensated for by the employment of women, who to date have been primarily responsible for family-related tasks (childcare and care of the elderly) (60). Thus, in recent years, a number of regulations have been adopted to support families (parental leave, right to day-care, etc.), and various institutional initiatives, such as the provision of care places or the offer of more flexible management of working time, have been initiated. Additionally, integration programs have been established to allow an easier transition back to professional life. However, the current measures are not sufficient to solve the problem, mainly because they have been comprehensively introduced and thus do not meet individual demands (61, 62).

The present study assessed the compatibility problem by means of the Work–Family Conflict Scale and the Family–Work Conflict Scale (24). The cut-off values of WFC and FWC were not exceeded by the total sample; however, more than a third of the respondents (*N* = 813) scored higher than the cut-off values of WFC (35.3%) and FWC (39.3%). For both scales, significantly more men than women suffered from compatibility conflict. WFC and FWC are higher for the male than for the female respondents, with more men scoring higher than the cut-off values of WFC and FWC (40.2% of *N* = 331 and 44.6% of *N* = 452, respectively) than women (31.4% of *N* = 452 and 35.4% of *N* = 446, respectively). Men suffered more frequently from feelings of helplessness in their situation. This may be due to the problem that young fathers, in particular, find it challenging to effectively take their parental leave without any career-related losses in exchange for this “timeout.” Additionally, women tend to work part-time more often and potentially perceive the interrole-conflict, particularly, the work–family conflict, as less stressful (63). This still poses a challenge even though increasingly more men and women wish to move away from traditional role models. Moreover, with generation Y, a new generation of fathers emerged who wish to spend more time with their family and who demand a better work-life balance. Studies on the age cohort generation Y indicate a lower willingness to work overtime or long hours or to sacrifice oneself for the job and instead display a shift toward a more equal work–life balance and a demand for the possibility to make work and family life realistically compatible (64).

The stress parameters analyzed (overtime is stressful, occupational stress (ERI ratio) and overcommitment) all correlate with the interrole conflict parameters, WFC and FWC, and with the health parameters physical and mental health and burnout. These correlations indicate that successful compatibility of work and family with a low conflict potential has positive implications for the mentioned occupational stress and health parameters and vice versa.

To assess the predictors for the incidence of work–family conflict and family–work conflict, multiple regression analyses were conducted. The inclusion criteria for the independent variables were the significant correlation analyses conducted beforehand. Additionally, demographic items were included. Both regression analyses revealed that the factors “stress due to overtime” and “gender” (male) served as the main predictors for a conflict between work and family. The regression analysis for work–family conflict (WFC) (the conflictual influence of work on the familial life), illuminated the burnout factor. The main predictors were “emotional exhaustion,” “sample” (namely the university hospital), as well as “stress due to overtime” and “gender” (male). Further, “occupational area” and “overcommitment” were calculated as additional predictors for WFC. Predictors for the family–work conflict (FWC) (the conflictual influence the family has on work life) are “children,” “overtime,” “age” (young people are more affected), and “gender” (male). This finding is in line with previous analyses by Niessen et al. (65), Ford et al. (66), and Demerouti et al. (67). Based on the literature, it is known that the compatibility conflicts (WFC, FWC) correlate with occupational stress (68), burnout (68), and depression (24, 69). Another study describes time pressure as a mechanism that, when employees become parents, may lead to additional psychological stress due to the double burden that parents face (70). The present study could also identify occupational stress in terms of stressful overtime and burnout (emotional exhaustion) as a predictor for the conflict between work and family. Also, both compatibility scales correlated significantly with occupational stress and overcommitment as well as with the health parameters physical and mental health. If one were to create a “typical” profile for a university employee at risk of experiencing a family–work and, respectively, work–family conflict, if would be a male, already emotionally exhausted employee of the university hospital, stressed by extra work, children, and the tendency to overcommit. This allows the conclusion that the focus of future support for more compatibility of work and family should be directed increasingly toward men with children. Additionally, besides preventive measures, this support should include individual behavior-preventive measures.

How the compatibility between work and family can be improved, particularly at universities, was evaluated by means of a demand analysis within the course of the current study; however, it is not the central subject of this publication.

Compatibility conflicts should constitute a central aspect of future orientations of institutions such as colleges. This can be confirmed by a study by Ernst and Young (71), which interviewed 3500 students on their wishes with regard to their potential future employers: 49% of the female and 29% of the male interviewees named the compatibility of work and family as an important criterion. Demographic changes and the necessity for an increase in female labor participation foster these demands. For universities, family-friendly environments are an important factor in employee loyalty and are thus a key success factor for the competition between the institutions for students and employees.

Limitations: The results described in this study, and particularly the predictors, have been assessed merely on the basis of a cross-section study. The initial intention of a longitudinal study could, to date, not be followed up, even though different calculations, such as regression analyses, and different scientific questions, such as the development of occupational stress and psychological stress over time, could be better substantiated and more exactly addressed in a longitudinal design. Since our study was conceptually designed with a longitudinal design and the intention of evaluating the changes that have already occurred, a follow up is urgently necessary. Regarding the recruitment of the sample, it was unfortunately not possible to recruit all the physicians employed at the university hospital, but only the doctors employed at the medical faculty with a contract with the state. Also, the nursing personnel could not be interviewed. For future follow-ups, it would be desirable to integrate all employees of the university hospital into the survey. Another limitation of the cross-sectional design is the fact that regression analyses could not be calculated with longitudinal data. Age as an influencing factor could not be statistically controlled for, since the requirements by the staff council did not allow any detailed demographic information. For this reason, no precise comparisons between the faculties or specialists or departmental levels could be carried out. Additionally, in the present publication, no detailed comparison between the occupational groups could be computed due to limited space. Since no demographic characteristics were available due to data protection, the present data cannot be tested for representativeness.

The results of the current study indicate a notable need for action regarding the work–life balance and compatibility of work and family for university employees. Especially in connection with the field of occupational stress and health, the results form a solid basis for the starting points of job-specific prevention and intervention measures. To this end, the planned longitudinal survey should urgently be conducted in a follow-up.

## Data Availability Statement

The raw data supporting the conclusions of this article will be made available by the authors, without undue reservation, to any qualified researcher.

## Ethics Statement

The authors assert that all procedures contributing to this work comply with the ethical standards of the relevant national and institutional committees on human experimentation and with the Helsinki Declaration of 1975, as revised in 2008. All study participants gave their written informed consent for inclusion.

## Author Contributions

LJ-B and KL-E: conceptualization. LJ-B: statistical analysis. LJ-B and SW: methodology. LJ-B, PB, and JS: writing—original draft preparation.

### Conflict of Interest

The authors declare that the research was conducted in the absence of any commercial or financial relationships that could be construed as a potential conflict of interest.
